# Optimizing performance of GATK workflows using Apache Arrow In-Memory data framework

**DOI:** 10.1186/s12864-020-07013-y

**Published:** 2020-11-18

**Authors:** Tanveer Ahmad, Nauman Ahmed, Zaid Al-Ars, H. Peter Hofstee

**Affiliations:** 1 Accelerated Big Data Systems Group, Quantum & Computer Engineering Department, Delft University of Technology, Delft, The Netherlands; 2 IBM Research Austin, Texas, USA

**Keywords:** Genomics, Whole Genome/Exome Sequencing, Big Data, Apache Arrow, In-Memory Data, GATK Best Practices

## Abstract

**Background:**

Immense improvements in sequencing technologies enable producing large amounts of high throughput and cost effective next-generation sequencing (NGS) data. This data needs to be processed efficiently for further downstream analyses. Computing systems need this large amounts of data closer to the processor (with low latency) for fast and efficient processing. However, existing workflows depend heavily on disk storage and access, to process this data incurs huge disk I/O overheads. Previously, due to the cost, volatility and other physical constraints of DRAM memory, it was not feasible to place large amounts of working data sets in memory. However, recent developments in storage-class memory and non-volatile memory technologies have enabled computing systems to place huge data in memory to process it directly from memory to avoid disk I/O bottlenecks. To exploit the benefits of such memory systems efficiently, proper formatted data placement in memory and its high throughput access is necessary by avoiding (de)-serialization and copy overheads in between processes. For this purpose, we use the newly developed Apache Arrow, a cross-language development framework that provides language-independent columnar in-memory data format for efficient in-memory big data analytics. This allows genomics applications developed in different programming languages to communicate in-memory without having to access disk storage and avoiding (de)-serialization and copy overheads.

**Implementation:**

We integrate Apache Arrow in-memory based Sequence Alignment/Map (SAM) format and its shared memory objects store library in widely used genomics high throughput data processing applications like BWA-MEM, Picard and GATK to allow in-memory communication between these applications. In addition, this also allows us to exploit the cache locality of tabular data and parallel processing capabilities through shared memory objects.

**Results:**

Our implementation shows that adopting in-memory SAM representation in genomics high throughput data processing applications results in better system resource utilization, low number of memory accesses due to high cache locality exploitation and parallel scalability due to shared memory objects. Our implementation focuses on the GATK best practices recommended workflows for germline analysis on whole genome sequencing (WGS) and whole exome sequencing (WES) data sets. We compare a number of existing in-memory data placing and sharing techniques like ramDisk and Unix pipes to show how columnar in-memory data representation outperforms both. We achieve a speedup of 4.85x and 4.76x for WGS and WES data, respectively, in overall execution time of variant calling workflows. Similarly, a speedup of 1.45x and 1.27x for these data sets, respectively, is achieved, as compared to the second fastest workflow. In some individual tools, particularly in sorting, duplicates removal and base quality score recalibration the speedup is even more promising.

**Availability:**

The code and scripts used in our experiments are available in both container and repository form at: https://github.com/abs-tudelft/ArrowSAM.

## Introduction

The **genome** of an organism is the complete set of its genetic material represented by its DNA sequence. Each cell in a human body contains a complete replication of the approximately 3 billion base pairs (bps) of DNA. The **genomics** field emphasizes on the understanding of structure, mapping and function of individuals genes (the genome) to get insights into their interaction and evolution with respect to one’s environment. In **comparative genomics**, complete genome features of different spices are extensively compared (for example with a reference genome) using computational tools. These comparisons can lead to fully characterize the resemblances and differences in one’s genomic features, trace down their origin or lineage, how the change or loss emerges throughout the evolutionary lineages and discover ways to cure diseases caused by genetic variations and developing personalized medicine and improving environmental health [1].

**Variant calling** is indispensable for comparative genomics as it reveals deep insights into nucleotide-level organismal differences in some specific traits among populations from an individual genome sequence data. Variant calling discerns genetic variations in three categories like, single nucleotide polymorphisms (SNPs), insertions and deletions (indels), and/or structural variants (SVs, may also include Copy Number Variants (CNVs), duplication, translocation, etc). An **SNP** reports a single base change in two genomes while the DNA around that base remains unchanged. **Indels** are single bases which have been inserted, or deleted in a genome when aligning to another reference genome. **Structural variants** are observed in organism’s chromosome structures. Generally defined as a region of DNA approximately 1 kbp or larger in size having variations in the form of inversions, translocations or deletions, insertions and CNVs (also called duplications). DNA sequencing reveals that CNVs are commonly observed in various organisms, particularly in human, which vary from individual to individual. Approximately two third of whole human genome is composed of such repeats.

DNA can mutate in any of the somatic cells or in germinal cells (germ cells); such variations are referred as somatic and germinal mutations, respectively. **Somatic analysis** identifies the variations in normal and tumor affected tissues. Somatic mutations/variations can cause cancer or other diseases. In **germline analysis** the variations in an individual’s DNA inherited from parents are analyzed to identify presence of inherited disease.

In **whole-genome sequencing (WGS)** the complete set of DNA sequences (both the entire protein coding and the non-coding regions of the genome) of an organism are determined. This gives a comprehensive and precise fingerprint of the whole DNA. **Whole-exome sequencing (WES)** instead just focuses on collecting DNA sequences of some specific regions (like protein coding). WES samples are typically sequenced at 100X or 30X coverage which focuses on less than ∼5% of the complete genome. Both techniques have their own benefits. WES saves costs and also gives more DNA coverage resulting in higher accuracy. WGS covers the complete genome which is good for fully characterizing and understanding the genome.

### Genome/Exome pre-processing

The pre-processed genomics data can be used for timely identification of gene mutation, diagnosis of disease as well as the development of targeted therapies. Generally pre-processing steps include alignment, sorting and duplicate reads removal from target genome sequence data. Many tools have been developed for analysis of high throughput sequencing data, from local alignment database search tools like BLAST [2], FASTA [3] to pairwise alignment tools like MALIGN [4], EMBOSS [5], tools like BLAT [6] Bowtie2 [7] and BWA [8] for short read sequence alignments and Minimap and Miniasm [9], DALIGNER [10] and DARWIN [11] tools for long reads alignment and mapping. Tools like SAMTools [12], Picard [13], Sambamba [14] and samblaster [15] are developed for alignment post-processing stages like indexing, sorting, duplicates removal in SAM/BAM (Binary Alignment/Map) files.

### Variant callers

GATK and Freebayes are commonly used open-source tools for germline variant calling analysis. Tools like VarScan [16], VarDict [17], MuTect2 [18] are used for somatic variant calling analysis. FreeBays [19], SNVer [20] and LoFreq [21] are also used for both germline and somatic variant calling analysis. Pisces [22] and Strelka2 [23] are recently developed open source tools by Illumnia for short variant calling to analyze both germline and somatic variations. DeepVariant [24] is deep convolutional neural network based variant caller. Both Strelka2 and DeepVariant variant callers outperform GATK, FreeBays and samtools in PrecisionFDA (pFDA) Challenges for precision and accuracy on indels and SNVs for different data sets. The output of these tools is generated in the variant calling format (VCF) to visualize and further analyze the detected variations.

### Challenges in genomics data processing

Comparative genomics is a young field. To process and analyze genomics data, the research community is actively working to develop new, efficient and optimized algorithms, techniques and tools, usually programmed in a variety of languages, such as C, Java or Python. As we have mentioned earlier, in order to construct a whole workflow for complete genome analysis, one has to use a combination of different open-source tools. These tools share the following common characteristics that impose limitations on the performance achievable by the genomics workflow.

- These tools are developed to use traditional I/O file systems, which incur a huge I/O bottleneck in computation due to disk bandwidth [25]. Each tool reads from the I/O disks, computes and writes back to disk.
- Due to the virtualized nature of some popular languages used to develop genomics tools (such as Java and Python), these tools cannot exploit modern hardware features like multi-core parallelization, Single instruction, multiple data (SIMD) vectorization and accelerators (like GPU or FPGAs) performance very well.
- In between processes data communication developed in different languages, a huge (de)-serialization and copy overheads incur.

### Motivation

New storage-class memory (SCM) technologies will soon replace the existing long latency and block-based data transfer HDDs/SSDs storage. Intel’s phase-change memory (PCM) based Optane DC (Data Center) Persistent Memory is one of the first candidates in this paradigm to accelerate big data workloads for in-memory analytics and provide fast startup-times for legacy applications/virtual machines in cloud environments [26–28]. Using these memories to store SAM data in columnar format and shared memory objects can provide benefit in many aspects to improve overall system throughout:

- One is related to the tabular nature of genomics data (SAM) in-memory.
- Second is related to underlying hardware technology to exploit the maximum cache spatial locality and SIMD vectorization capabilities of modern multi-core systems.
- Third is to avoid (de)serialization of data when processing in different languages. Shared memory objects of SAM data can be processed in parallel.

We use DRAM as an alternative to such memory technologies for evaluation purpose because of its same characteristics of byte-addressability (load/store access to memory) and lower latency.

## Background

This section provides a short description of widely-adopted GATK variant calling workflow, NGS technologies and the amount of data they produce and the challenges in processing this data. A brief introduction to the Apache Arrow framework and its Plasma shared memory API is also given.

### Genome Analysis Toolkit (GATK)

GATK [29] from the Broad Institute is considered as a benchmark for variant calling discovery. As the SAM [30] is a de-facto format for storing NGS data, its compressed and indexed BAM [30] version is used in GATK tools as input file(s). GATK tools produce variant calling outputs in many different formats like VCF, GVCF and different useful statistics in text format. GATK internal architecture is based on the philosophy of MapReduce [31] functional programming paradigm to achieve maximum parallel efficiency by distributing data among processes. In MapReduce programming, the computations are accomplished in two steps; first the problem is divided into many discrete independent tasks which are fed to the map function. After completion of tasks their respective outputs are merged into a reduce function to generate a final output product. GATK reads/writes data files through htsjdk library, divides and prepares data in traversals then processes in walker modules. The walker modules provide the map and reduce functions for data consumption [32].

### GATK best practices workflows

For the analysis and interpretation of NGS data to be used in clinical settings, different tools and workflows have been created. GATK recommended best practices for variant calling proposes BWA-MEM for mapping reads, while Picard or Sambamba can be used for sorting and mark duplicates removal in the reads. Base Quality Score Recalibration (BQSR) in GATK adjusts the quality score of reads by employing machine learning algorithm. The following common GATK workflows [33, 34] are available in GATK4 for different types of variant calling. 1. For identifying germline short variants (SNPs and indels) in one or more individuals the Haplotypecaller algorithm is used to generate a joint callset in VCF format. 2. Similarly Mutect2 is used for somatic short variants (SNVs and indels) identification in one or more tumor samples in a single individual, with or without a matched normal sample. 3. For germline short variants (SNPs and indels) discovery in human exome sequencing data the workflow uses intervals file in BED format while the Haplotypecaller algorithm is used to generate a joint callset in VCF format.

### Next-generation sequencing: technologies and data

The first ever Human Genome Project [1990—2003] concluded an initial sequence draft of human genome consisting of approximately 2.85 billion nucleotides [35]. Since then, genomics data has been increasing rapidly due to the innovations in genome sequencing technologies and analysis methods. Second Generation Sequencing (NGS) technologies like Illumina’s HiSeqX and NextSeq produce whole genome, high throughput and high quality short read data at a total cost of <DOLLAR/>1K per genome, which is expected to drop down below <DOLLAR/>100 for more advanced sequencing technologies. Third generation sequencing technologies are now capable of sequencing reads of more than 10 kilo-base-pairs (kbp) in length, such as Oxford Nanopore, Single Molecule Real-Time and Pacific BioSciences sequencing technologies. The ongoing pace of these technologies promises even more longer reads of ∼100 kbp on average. Long reads produced by third generation sequencing technologies provide the prospect to fully characterize genomes at high resolution for precision medicine [36].

### Apache Arrow

**Fig. 1:**
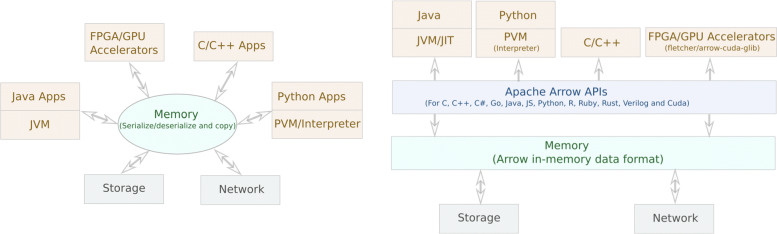
**Left:** An example where (de)serialization and copy takes place when data is exchanged between different languages and platforms. **Right:** Apache Arrow provides a unified in-memory format for data placement which can be used in many languages and platforms avoiding the (de)serialization and copy overhead

The Apache Arrow [37] project was initiated by the Apache Foundation in 2016. This framework provides an open and a common standardized format for different programming languages for reading/writing tabular data in-memory. Through language-specific libraries, multiple languages can share data without any copying or serialization. This in-memory access of data through Apache Arrow is illustrated in Fig. 1. At the time of writing, Apache Arrow supports the following languages: Go, C, C++, C#, Java, JavaScript, R, Rust, MATLAB, Ruby and Python. Interfaces exist for GPGPU programming, through Arrow CUDA interfaces. External tools to support FPGA accelerators also exist through the Fletcher project [38]. In the Arrow format, data entries (records) are stored in a table called a RecordBatch. Each record field is stored in a separate column of the RecordBatch table in a manner that is as contiguous as possible in memory. This is called an Arrow Array which can store data of different types—i.e., int, float, strings, binary, timestamps and lists, but also nested types (such as lists of lists, etc.). Arrays may have different types of physical buffers to store data. This layout provides higher spatial locality when iterating over column contiguous data entries for better CPU cache performance. SIMD (Single instruction, multiple data) vector operations can also benefit from such a layout as vector elements are already aligned properly in memory.

### Plasma in-memory object store

Plasma is an inter-process communication (IPC) component of Arrow, that handles shared memory pools across different heterogeneous systems [39]. To perform IPC, processes can create Plasma objects inside the shared memory pool, that are typically data buffers underlying an Arrow RecordBatch. Through the shared memory pool, Plasma enables zero-copy data sharing between processes.

## Implementation

**Fig. 2:**
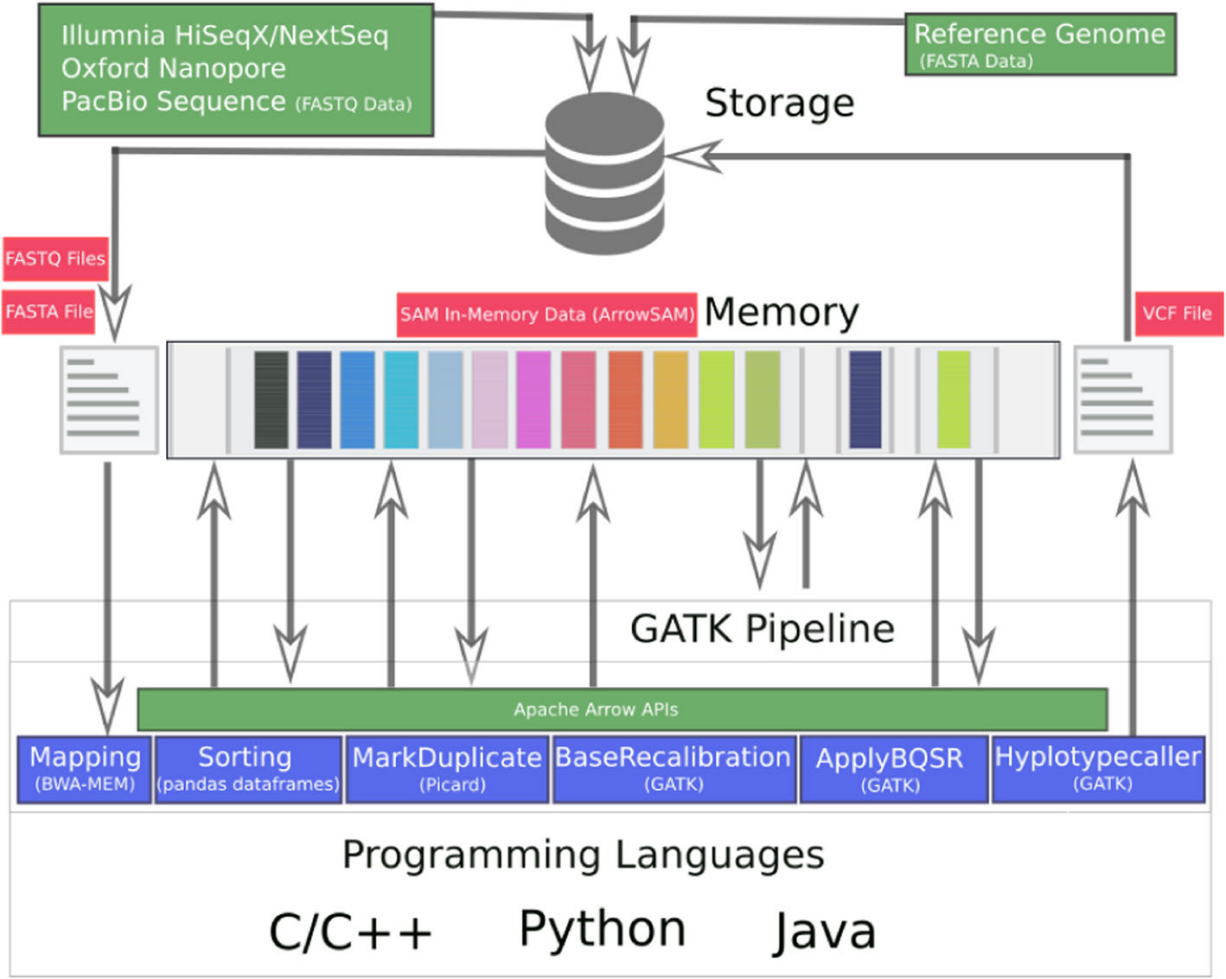
In-memory architecture of GATK best practices recommended workflow using Arrow in-memory SAM representation for all intermediate steps

**Fig. 3:**
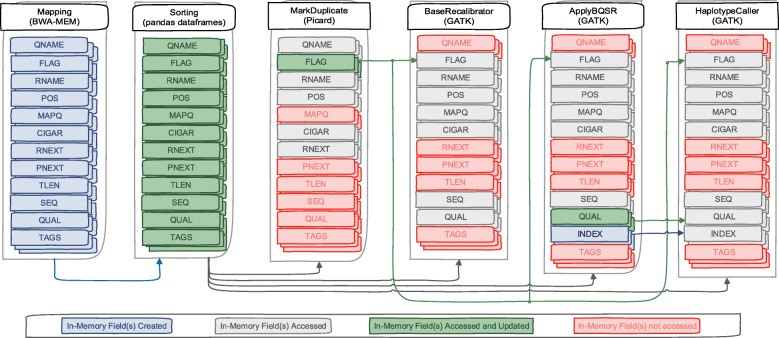
In-memory SAM data placement for all chromosomes (1-22, X, Y and M) in GATK best practices workflow. Applications access it through shared memory plasma objects. For higher sequencing coverage data i.e, WGS data, each chromosomes with size more than 2GB are further divided for scalability

In order to enable genome pre-processing applications and GATK (Fig. 2) to use in-memory SAM data, two main optimization are required. First, we need to define an in-memory Arrow representation of the SAM data. Second, the applications need to be adapted to access the new in-memory SAM data. These applications access, update and create new data fields as shown in Fig. 3. In the following, these two optimizations are discussed.

### In-memory SAM format

The SAM file format is an ASCII based, tab delimited text format to represent DNA sequence data. We create an in-memory SAM representation using the Apache Arrow columnar format that consists of the same fields (columns) used in SAM to store the corresponding data, this format is also explicitly explained in our previous work [40]. We call this the ArrowSAM format, this stores the data in RecordBatches. Each RecordBatch is a combination of a schema, which specifies the types of data fields of ArrowSAM and the data itself, more details of in-memory Arrow data representation can be found in [40].

Genomics applications can use ArrowSAM to create RecordBatches of genomics data in-memory.

RecordBatch columnar data can be deleted/updated in the same application but to make data usable in other applications we have to use shared memory flat buffers.

### BWA-MEM integration

BWA-MEM is the most popular alignment algorithms in the bioinformatics community due to its efficient and accurate alignment of raw FASTQ data against a large reference genome. After performing alignment of each read, it creates a SAM record of twelve data fields as shown in Fig. 3. Instead of writing these records in a SAM file, we modified BWA-MEM to use the ArrowSAM format and Arrow libraries to store these records in Arrow Buffers. We have created as many such buffers as number of chromosomes. So we check the reference name (RNAME) of each record and insert to its respective buffer. At the end of the alignment process, all the buffers are converted to RecordBatches which are inserted into shared memory pool.

### Sorting through pandas dataframes

Randomly generated SAM reads need to be sorted by their respective chromosome and individual coordinates (begin positions) within a chromosome. Pandas is a powerful and easy to use python library, which provides data structures, data cleaning and analysis tools. Dataframes is an in-memory data library that provides structures to store different types of data in tabular format to perform operations on the data in columns/rows. Any row in a dataframe can be accessed with its index, while a column can be accessed by its name. A column can also be a series in pandas. Using dataframes with python arrow bindings (PyArrow) illustrates the powerful capabilities of in-memory data representation. Tools like Picard, Samtools and Sambamba are used to sort the reads in a SAM file according to the chromosome name and start positions of each read. This type of sorting becomes computationally intensive when the whole SAM file needs to be parsed and sorted based on these two fields. In contrast, our implementation uses pandas dataframes to sort each individual chromosome based on the start position of reads in that particular chromosome. This reduces the computational effort needed to sort the reads since we already assign them to the RecordBatch that belongs to their own chromosome. Therefore, we only need to sort them based on their position. We create new RecordBatches for each chromosome with sorted data as shown in Fig. 3.

All shared memory objects of chromosomes are fed to pandas dataframes to sort in parallel. After sorting, the new sorted chromosomes RecordBatches are stored in shared memory by deleting previous shared memory objects, to be used by subsequent applications.

### Picard MarkDuplicate integration

After sorting the reads by their coordinates, the duplicate reads with low quality should be removed. The MarkDuplicate tool in the Picard package is considered as a standard algorithm for duplicate reads removal. This tool reads the SAM files two times, first when building the sorted read end lists and second when removing marked duplicates from those lists by comparing each individual read in the file. This tool has two main limitations: first it reads SAM data sequentially from the input file, second it converts all input file reads data into their corresponding SAM records. SAM files are usually stored in disk in compressed format (called BAM) that has a compression ratio of about 30%. This means every time we read and write these files to disk, we have to incur a the overhead of compression and decompression. To overcome these overheads, we just read the data as ArrowSAM format in-memory once, accessing only five fields (QNAME, FLAG, RNAME, POS, CIGAR and RNEXT) as shown in Fig. 3, which are actually needed to perform the MarkDuplicate operation. For this purpose, We have modified the htsjdk (a java API used in Picard, GATK and many other tools for managing I/O access of high-throughput sequencing data files) to access shared memory stored plasma objects, and parse them to their respective RecordBatch. Each SAM read with the above mentioned five fields is accessed via index. Each shared memory object contains one chromosome SAM data. To take advantage of this, our implementation processes all chromosomes in parallel by initiating as many Picard instances as number of chromosomes. After processing reads, MarkDuplicate sets the duplicate bit in the FLAG field, so only the FLAG field is updated in this process which is written in a separate shared memory object for each chromosome. After completion of the MarkDuplicate stage, the sorted and updated duplicate flag data is available in shared memory objects for further analysis.

### GATK BaseRecalibration integration

Variant calling heavily relies on the assigned base quality scores per base in individual reads. These scores are estimates of sequencing machine errors in producing bases. However, these scores are also affected due to systematic errors in the sequencing machines. BaseRecalibration finds systematic error patterns by analyzing how these errors vary over all bases. Only seven fields are accessed: six fields (RNAME, POS, MAPQ, CIGAR, SEQ and QUAL) from ArrowSAM records of shared memory objects created in the ’Sorting’ process, and one (FLAG) field created in MarkDuplicate process as shown in Fig. 3. We have also modified the access to the htsjdk library for this application similar to the MarkDuplicate application. All shared memory objects of individual chromosomes are processed in parallel by initiating as many as BaseRecalibration instances as number of chromosomes. All relevant information generated by this tool is recorded in tables.

### GATK ApplyBQSR integration

ApplyBQSR applies numerical corrections to each individual basecall based on the patterns identified in BaseRecalibration tables. This application generates new QUAL and INDEX fields which are written in a separate shared memory object. In this application, the same seven fields are accessed: six fields (RNAME, POS, MAPQ, CIGAR, SEQ and QUAL) from in-memory SAM records of shared memory objects created in ’Sorting’ process and one (FLAG) field created in MarkDuplicate process as shown in Fig. 3. We have also modified htsjdk library for this process similar to previous processes except in generating output. Because we limit our processing to specific parts of the genome by filtering out unused intervals as provided in a special filtering file (called bed file), only those reads which fall in these specific intervals are forwarded for further processing. To properly map the newly created QUAL field output with that of original in-memory ArrowSAM data of the sorting process (to be used in the next application), we have appended an additional field ’index’. This field stores the index of the original read. All shared memory objects of individual chromosomes are processed in parallel by initiating as many as ApplyBQSR instances as the number of chromosomes.

### GATK HaplotypeCaller integration

HaplotypeCaller calls SNPs and Indels through local de-novo assembly in active regions. Active regions are those which have some sufficient probability of variation. Here eight fields are accessed: five fields (RNAME, POS, MAPQ, CIGAR and SEQ) from ArrowSAM records of shared memory objects created in the ’Sorting’ application, one (FLAG) field created in the MarkDuplicate application and two (QUAL and INDEX) field created in the ApplyBQSR application as shown in Fig. 3. We have also modified access to the htsjdk library for this process similar to previous processes like ApplyBQSR. First, the INDEX field is checked as an alternative to intervals, so that particular index in the original ArrowSAM object created in the ’Sorting’ process is accessed. This indexing technique has one benefit and also one drawback. In terms of benefit, using the index field, we access only those fields which fall in given bed file intervals for exome analysis. Drawback is related to cache performance. Due to repeatedly changing the index during reads access, the cache spatial locality cannot be exploited efficiently. The output of this process is generated in VCF file format. Because we are processing all the shared memory objects of individual chromosomes in parallel, separate chromosome files are generated which need to be merged for further variants analysis.

## Methods

We compare our ArrowSAM-based workflow to a number of popular workflows used in the field. For alignment we use BWA-MEM for all workflows due to its high accuracy and efficiency. For sorting and duplicate removal, Picard, Sambamba and elPrep (sfm) have been used. GATK and elPrep are used for the base recalibration and variant discovery stages. The reason behind selecting elPrep for performance comparison is the fact that it uses in-memory, and multi-threading techniques for pre-processing and variant discovery, while reporting to produce the same accuracy as that of GATK [41]. In contrast, our implementation also facilitates in-memory and multi-threading features while using the exact same Picard and GATK applications. The reason for selecting Sambamba for the comparison is its multi-threaded nature and for being more efficient than other open source tools available for sorting and mark duplicate operations with the same accuracy as Picard.

In the following subsections, we discuss the workflows used in comparison to ArrowSAM based implementation.

### Storage (BWA-MEM - Picard - GATK)

This combination of tools is used in almost all GATK recommended best practices workflows for both whole genome and whole exome sequencing analysis. Both reference and query raw data sets are placed in local storage and all applications access data through local disk I/O. All the immediate results of each application are also written in local disk in standard SAM/BAM files.

### Storage (BWA-MEM - Sambamba - GATK)

Sambamba is used here as an alternative to Picard for sorting and mark duplicates operations. Sambamba as mentioned earlier is faster than Picard for both of these applications because of multi-threading. But unfortunately parallel performance of Sambamba is limited and not scalable due to I/O saturation. All data sets and immediate results of each application are using local storage for I/O.

### ramDisk (BWA-MEM - Sambamba - GATK)

In this workflow, we use ramDisk (memory-mapped disk) instead of local storage, since we can improve performance of these applications by placing data closer to the processor. This way, all data sets and immediate results of each application are kept in ramDisk in standard SAM/BAM files.

### ramDisk (BWA-MEM - Sambamba - GATK (Parallel))

We can use some sort of naive parallelism for performance improvement in some GATK applications. For example in whole exome sequencing, BaseRecalibration application uses an interval file with -L option. If we split the interval file for each chromosome and pass the individual interval files to multiple instances of the BaseRecalibration application each executed for an individual chromosome in parallel, it will generate output ‘tables’ separate for each chromosome. Then, ApplyBQSR can also use the individual chromosomes interval files and ‘tables’. So running the ApplyBQSR instances in parallel will generate new BAM files for each individual chromosome separately. These individual chromosome BAMs and interval files can be passed to parallel instances of HaplotypeCaller, which will generate separate VCF files for individual chromosomes. These VCF files can then be merged in GATK.

### ramDisk (BWA-MEM - Sambamba (Pipes) - GATK (Parallel))

We can use Unix pipes in some intermediate applications to redirect their standard output to other application in the workflow as their input to save the I/O time and disk resources of local storage. Using Unix pipes, the output of an application is not stored in disk, but is buffered in memory temporarily until it is consumed by the next application in the pipe. We also naive parallelism for performance improvement in some GATK applications as mentioned in above method.

### ArrowSAM

This is our implementation proposed by this paper which uses in-memory ArrowSAM format and shared memory plasma objects to exploit cache spatial locality and multi-core efficiency. 1) Alignment is done in BWA-MEM which has already multi-threading support and output ArrowSAM data is placed in shared memory objects in respective chromosomes boundaries, 2) followed by coordinates based sorting using Plasma dataframes on all chromosomes (chromosomes greater than 2GB in WGS data sets are further divided with zero-copy overhead) to run sorting algorithm in parallel. 3) Picard MarkDuplicate is then run on resulting shared memory data chunks in parallel creating new FLAG field in-memory. 4) GATK BaseRecalibration generates tables for all ArrowSAM data chunks in parallel, 5) ApplyBQSR creates QUAL field by running parallel on all ArrowSAM data chunks and finally HaplotypeCaller generates separate VCF files for each chromosome/chunk (in case chromosome greater than 2GB). These files are merged to generate a final VCF using GTAK for futher analysis.

### elPrep

As discussed earlier, elPrep [41] is a multi-threaded pre-processing tool to operate on SAM/BAM data in-memory. In this tool, sorting, duplicate marking and base quality score recalibration algorithms are optimized for parallel execution. This tool has two runtime options, one is sfm, which uses less memory as compare to the other one called filter, which uses a large memory pool for in-memory processing. As reported in the paper, this tool has the same accuracy for pre-processing of SAM/BAM data as that of the GATK recommended best practices workflow. Therefore, we included this workflow for speedup comparison with all other workflows as this tool is more closely related to our implementation in the context of multi-threading and in-memory data placement and execution. Finally, GATK HaplotypeCaller is used for variant calling.

## Results

For evaluation of our in-memory SAM format and Apache Arrow integration into BWA-MEM, Picard and GATK tools, we have created a number of different workflows using state-of-the-art tools and techniques in accordance with GATK best practices workflow for whole genome and exome sequencing. We have run all these workflows with their recommended settings. In the “Performance evaluation” section below we describe the measured performance, while we discuss the results in the “Discussion” section.

**Fig. 4:**
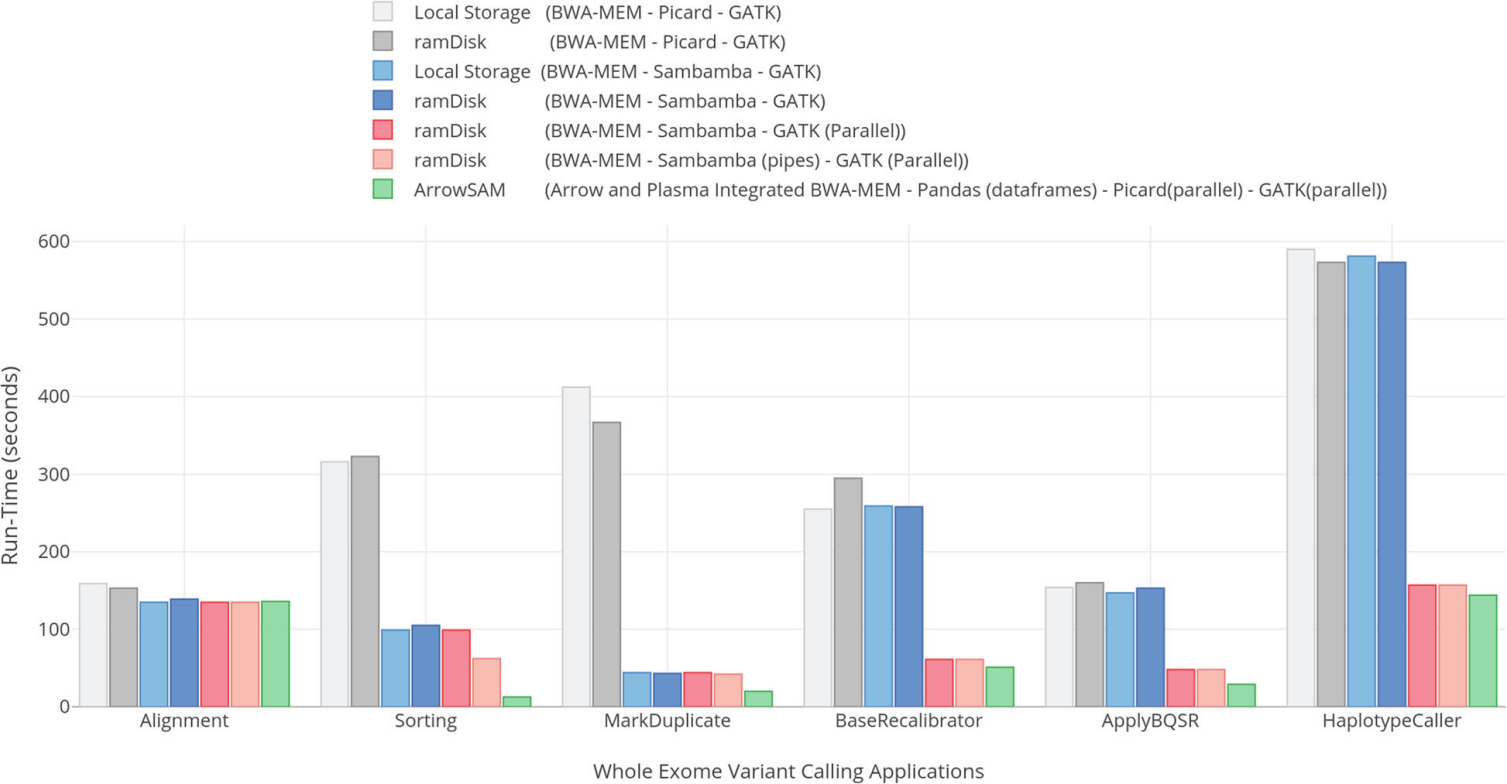
Runtimes (in seconds) of individual variant calling applications on whole exome data set using different workflow options (i.e, ramDisk, Pipes for Sambamba and chromosome wise parallelism in GATK)

**Fig. 5:**
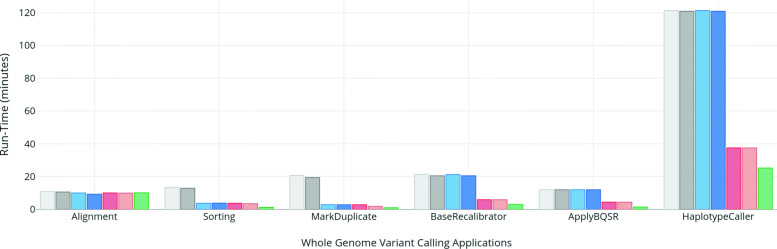
Runtimes (in minutes) of individual variant calling applications on whole genome data set using different workflow options (i.e, ramDisk, Pipes for Sambamba and chromosome wise parallelism in GATK)

**Fig. 6:**
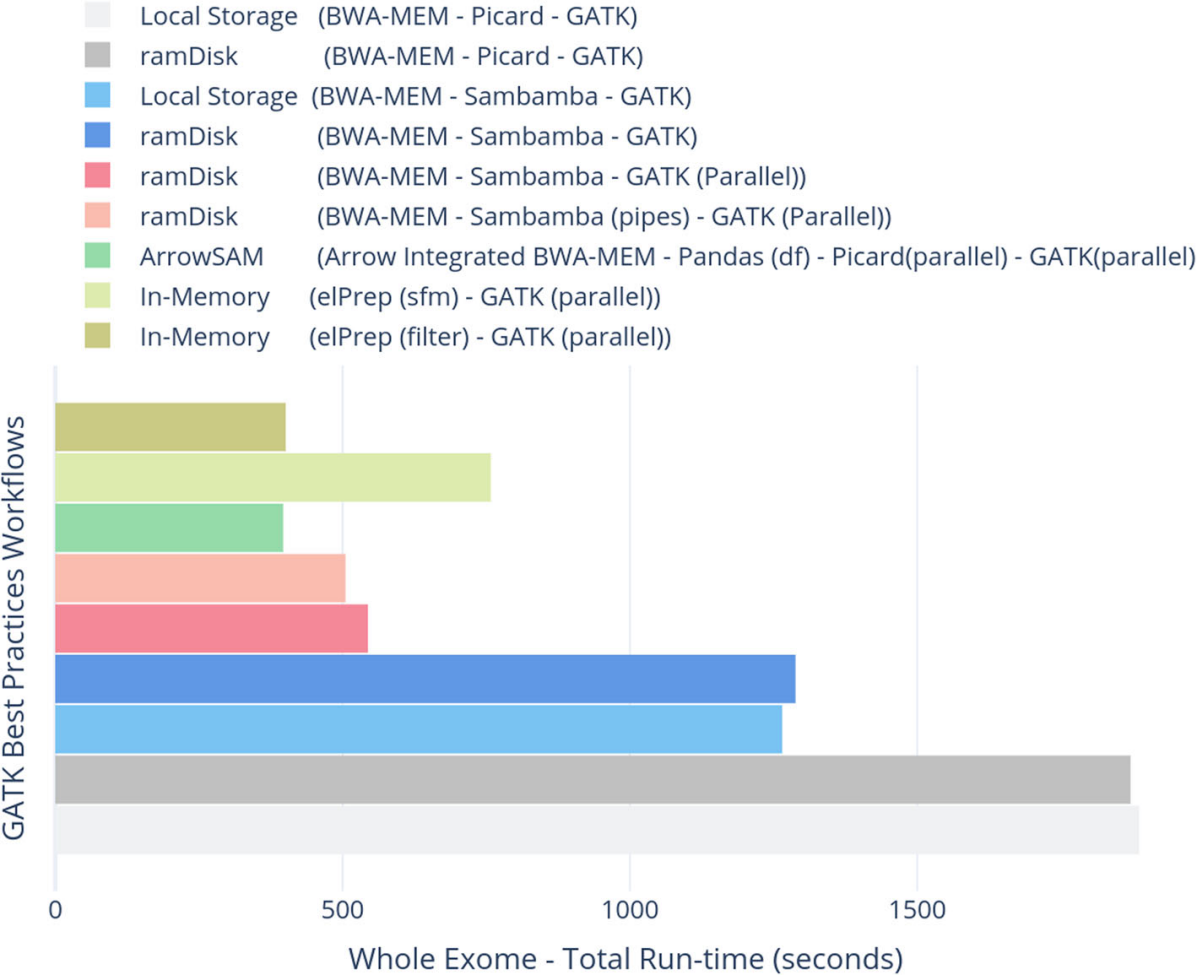
Total execution-times (in seconds) for complete variant calling workflows using different efficient options (i.e, ramDisk, Pipes for Sambamba and chromosome wise parallelism in GATK) on whole exome data set

**Fig. 7:**
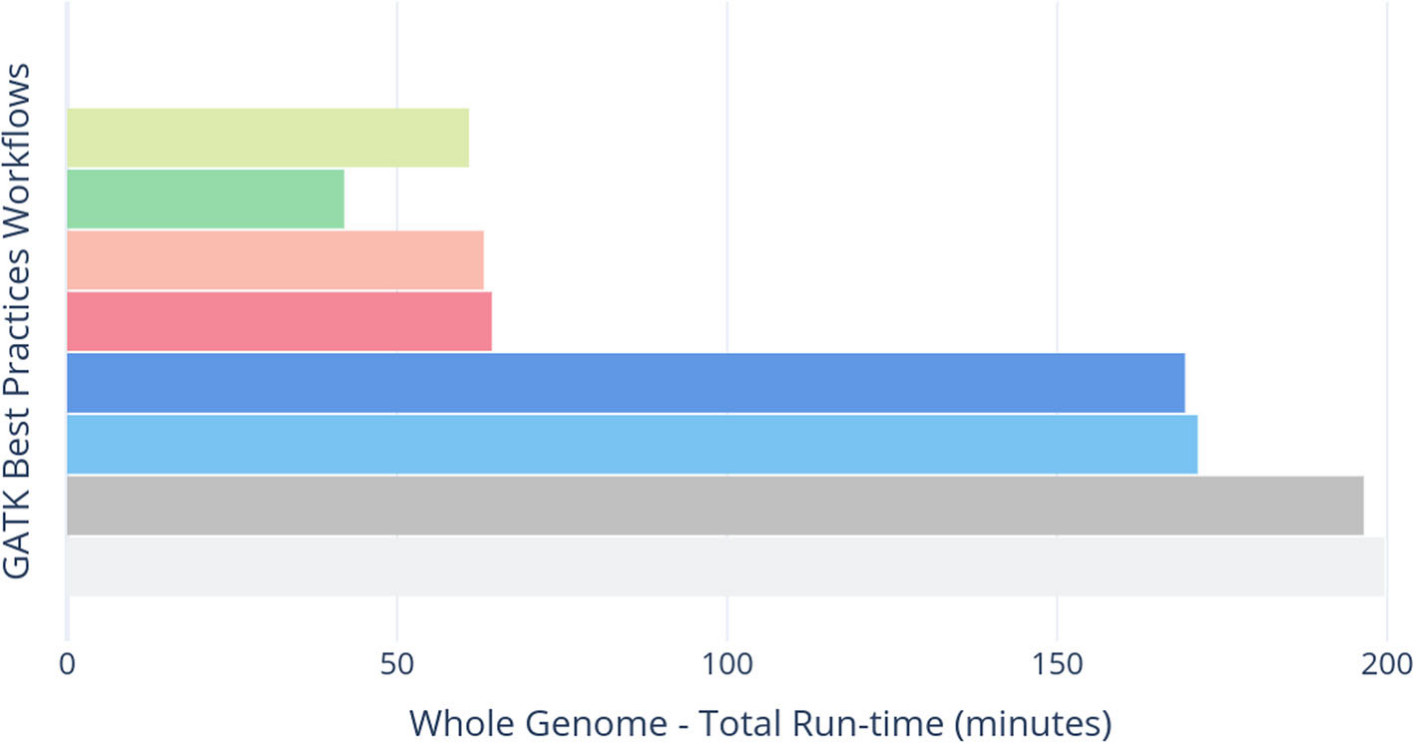
Total execution-times (in minutes) for complete variant calling workflows using different efficient options (i.e, ramDisk, Pipes for Sambamba and chromosome wise parallelism in GATK) on whole genome data set

The individual applications execution times of the various workflows for WES are shown in Fig. 4 while Fig. 5 shows the execution times for individual application for WGS. Similarly, the total execution times of the workflows are shown in Figs. 6 and 7 for WES and WGS data sets, respectively.

### Performance evaluation

In this section, we compare execution time of our GATK recommended best practices variant calling workflow using ArrowSAM with other state-of-the-art workflows as discussed in the “Methods” section on high throughput genome and exome data sets.

### Storage (BWA-MEM - Picard - GATK)

This represents the *baseline* workflow. The main performance bottleneck in this workflow is single-threaded disk I/O access of SAM/BAM file(s) by the htsjdk library which is used in Picard and GATK tools. This workflow takes highest runtime from pre-processing to variant calling among all workflows.

### Storage (BWA-MEM - Sambamba - GATK)

Replacing Picard with Sambamba for sorting and duplicate removal gives significant speedup in overall workflow execution as shown in Figs. 4 and 5. But Sambamba’s performance does not scale very well since increasing the number of threads above 12 for sorting and mark duplicate gives no performance improvement. Still, the individual time of sorting and mark duplicate is significantly less than the baseline with 12 threads resulting in an overall execution time speedup of 1.5x as compared to the baseline for whole workflow.

### ramDisk (BWA-MEM - Sambamba - GATK)

ramDisk is frequently suggested as an alternative for fast processing. We have observed that for GATK, only a small performance improvement is achieved. In the case of using ramDisk with Sambamba, there is even a reduction in performance for WES.

### ramDisk (BWA-MEM - Sambamba - GATK (Parallel))

Using ramDisk for Sambamba while running GATK in parallel for all chromosomes is able to achieve a better performance improvement as compared to all previous workflows. The speedup is 3.5x and 3x for WES and WGS data as compared to the baseline workflow, respectively.

### ramDisk (BWA-MEM - Sambamba (Pipes) - GATK (Parallel))

Redirecting output of BWA-MEM to Sambamba using Unix pipes slightly improves the performance of ramDisk. This is the best possible scenario of performance improvement as compared to previous workflows. It gives an overall speedup of 3.7x and 3.1x for WES and WGS data over the baseline workflow, respectively.

### ArrowSAM

ArrowSAM based workflow is the fastest among all workflows. This workflow is made as scalable as possible by employing widely used pre-processing and variant calling algorithms of Picard and GATK. We achieve a speedup of 4.76x and 4.85x for WES and WGS data in overall execution time as compared to baseline workflow, respectively. Compared to the fastest parallelized workflow (ramDisk (BWA-MEM - Sambamba (Pipes) - GATK (Parallel))), our ArrowSAM workflow achieves a speedup of 1.27x and 1.45x with WES and WGS data, respectively.

### elPrep

elPrep is a single application that can be used as a plug-in replacement for all pre-processing tools. That is the reason individual applications runtime is not shown in Figs. 4 and 5. When using the sfm option, elPrep gives a speedup of 2.49x and 3.26x over the baseline for WES and WGS data, respectively. However, it is 1.91x and 1.45x slower than ArrowSAM for WES and WGS data, respectively. This tool also has a filter option, which gives 4.7x speedup over the baseline and is only slightly slower than ArrowSAM for WES data at the expense of using 4x more memory than ArrowSAM. We are not able to run the filter option on WGS data due to large memory requirement. In the elPrep paper [41], the authors also do not show the results with the filter option for WGS data.

### Evaluation system

All experiments and comparisons are performed on a dual socket Intel Xeon Server with E5-2680 v4 CPU running at 2.40GHz. Each processor has 14 physical cores with support of 28 hyper-threading jobs. Both processors are connected through Intel QuickPath Interconnect and share memory through non-uniform memory access architecture. A total of 192-GBytes of DDR4 DRAM with a maximum of 76.8 GB/s bandwidth is available for the whole system. A local storage of 1-TBytes and the same amount of network attached storage is available on the system. CentOS 7.3 Minimal Server operating system is installed. All workflows are executed through bash scripts.

### Tools

The Apache Arrow framework and all its related libraries (like cglib, pyarrow and arrow-java) are installed in a Singularity container for ease of use to external users. The installed tools are listed in Table 1 with their versions for future reference.

**Table 1:** Tools and libraries used in the experimental setup

Tools/APIs	Version
BWA-MEM [8]	0.7.17
Picard [13]	2.18.14
GATK [29]	4.0.12.0
Sambamba [14]	0.6.8
elPrep [41]	4.1.5
Arrow C/C++/Java [37]	0.11.0
PyArrow [50]	0.11.0
Plasma Object Store [39]	0.11.0

### Datasets

We use Illumina HiSeq generated NA12878 dataset [42] with paired-end reads of WES of human with 30x sequencing coverage. Similarly for WGS, we use Illumina HiSeq generated NA12878 dataset sample SRR622461 with paired-end reads with sequencing coverage of 6x (we further lower the coverage to 2x due to memory limit on our evaluation system). Read length of 100 base-pairs is used for all data. Genome Human Genome Reference, Build 37 (GRCh37/hg19) is used as a reference genome. All workflows in our experiments use this data set for both WES and WGS.

### Memory footprint

Our implementation is solely memory based, so all the data between BWA-MEM and HaplotypeCaller applications remains in memory. We only compare runtime peak memory utilization of elPrep and ArrowSAM since in-memory resource requirements vary for intermediate operations. Table 2 lists the memory usage for both tools. elPrep uses almost the same memory as ArrowSAM on WES data with the sfm option enabled and uses 4x more memory with the filter option. For WGS data elPrep (sfm) and ArrowSAM have the same memory footprint but elPrep (filter) memory footprint is not available due to the large memory requirements beyond available system memory resources. This use case is also not covered in the original elPrep paper. The results show that ArrowSAM only uses memory that is comparable to the size of the SAM file for both WES and WGS data sets.

**Table 2:** Peak memory usage for in-memory processing tools

Tool	Exome	Genome
elPrep (sfm)	6.8GB	68GB
elPrep (filter)	26GB	X
ArrowSAM	7.2GB	69GB

### Discussion

Here we discuss some characteristics and limitations of our implementation in context of future perspective of in-memory data formats and processing for variant calling applications.

#### Parallelization and scalability

In ArrowSAM, all applications are capable to process data in parallel. The chunks of SAM data can be based on chromosomes or on the required data size. So that the memory plasma objects can be shared between different applications, which results in a large speedup in the overall runtime of individual applications.

#### CPU utilization

Depending on data partition in ArrowSAM, the maximum number of CPUs can be used for processing data in individual applications.

#### Cache locality

Due to the in-memory columnar data format, our implementation is able to exploit cache locality efficiently. All levels of cache accesses decrease in the sorting and mark duplicate applications, particularly due to the fewer number of in-memory fields (mostly integer type) access as discussed in [40]. Cache miss rate also decreases in all cache levels and particularly in level-1 cache. In BaseRecalibration, ApplyBQSR and HaplotypeCaller applications we also exploit cache locality but it is not much significant as compared to previous applications because of two reasons, 1. algorithms for these tools are not developed in such a way to exploit cache locality efficiently, and 2. base sequences (SEQ) and qualities (QUAL) fields are also being accessed which pollute cache lines early in these applications.

#### Accuracy

We did not change any part of actual algorithms in all Picard and GATK applications. Therefore, our results are exactly the same as in the original implementation of both tools.

### Code and scripts availability

The code for the in-memory ArrowSAM representation, all related Apache Arrow libraries for C, Java and Python languages and plasma shared memory process are installed on a singularity container which is freely available at https://github.com/abs-tudelft/ArrowSAM. The scripts for running all workflows are also available in the same root directory.

## Related work

Many in-memory workflows have been presented in the literature. Many of these implementations are cluster scaled and do not exploit single node performance taking advantage of the Apache Spark framework [43] for in-memory data management like SparkGA [44] and ADAM [45]. These implementations are not discussed in this paper. Our focus is to exploit the performance of single node systems.

Aginome IMP Platform [46], a GPU based sequence analysis tool set which uses in-memory database to store intermediate results for further analysis. To use in-memory database, the IMP Platform modifies FreeBayes [19] and GATK for use in variant calling. IMP with GATK speeds up the variant detection workflow by 30x, while IMP with FreeBayes improves the performance by 100x as compared to the BWA-GATK workflow. However, this tool is not open-sourced. The Sentieon Genomics Tools [47], report 10x performance improvement over GATK, MuTect and MuTect2 workflows by eliminating intermediate files merging. This tool also reports improving the performance of BWA-MEM by 1.9x times. However, this tool is also not available publicly and the paper does not discuss the details of the implementation.

elPrep [48], is a set of tools for pre-processing SAM/BAM files for variant calling. It is a multi-threaded, single command plug-in replacement tool which processes the data in-memory instead of reading and writing to I/O for each operation. In elPrep 4 [41], the authors reported 13x speedup over GATK best practices workflow for whole-exome and 7.4x speedup for whole-genome data using maximum memory and storage footprints, at the expense of excessive memory utilization. They also compare the results for a cluster deployment to show the scalability for high performance computing infrastructure. In memory-driven computing, a large pool of different types of memories are created and connected to the processing resources through the Gen-Z communication protocol. The memory is shared across the processes being executed to avoid intermediate I/O operations. This systems also allows byte-addressability and load/store instructions to access memory. [49] used a Gen-Z enabled platform for genomics and reported 5.9x speedup over the SAMtools baseline implementation for a number of DNA assembly algorithms. The source code is not available.

Some researchers use high-performance hardware accelerators such as GPUs [51] and FPGAs [52] to accelerate computationally intensive parts of genomics pipelines, but availability of such accelerators in the field remains limited.

## Conclusion

In this work, we integrate our Apache Arrow in-memory SAM representation (ArrowSAM) into genomics pre-processing and variant calling applications.

Our implementation shows that adopting in-memory SAM representation in genomics high throughput data processing applications results in better system resource utilization, low number of memory accesses due to high cache locality exploitation and parallel scalability due to shared memory objects. We compare a number of existing in-memory data placing and sharing techniques like ramDisk and Unix pipes to show how columnar in-memory data representation outperforms both. We achieve a speedup of 4.85x and 4.76x for WGS and WES data sets in overall execution time of variant calling workflows, respectively. Similarly, a speedup of 1.45x and 1.27x for these data sets is achieved, as compared to the second fastest workflow.

In future work, to feed processor fast and properly formatted data, in-memory data management techniques will be explored more rigorously to leverage the benefits of modern hardware features like multi-cores, vector units and to exploit caches locality in the presence of persistent memory technologies. We also plan to use ArrowSAM in big data frameworks like Spark for cluster level scalability of genomics applications.

## Data Availability

All the codes and scripts are publicly available at: https://github.com/abs-tudelft/ArrowSAM.

## References

[1] Xia X. Comparative Genomics; 2013. 10.1007/978-3-642-37146-2.

[2] Altschul SF, Gish W, Miller W, Myers EW, Lipman DJ (1990). Basic local alignment search tool. J Mol Biol.

[3] J Lipman D, Pearson W (1985). Rapid and sensitive protein similarity searches. Science (New York, N.Y.).

[4] Wheeler WC, S. Gladstein D. Malign: A multiple sequence alignment program. J Hered. 1994; 85. 10.1093/oxfordjournals.jhered.a111492.

[5] Rice P, Longden I, Bleasby A (2000). Emboss: The european molecular biology open software suite. Trends Genet TIG.

[6] James Kent W (2002). Blat - the blast-like alignment tool. Genome Res.

[7] Langmead B, Salzberg SL (2012). Fast gapped-read alignment with bowtie 2. Nat Methods.

[8] Li H, Durbin R (2009). Fast and accurate short read alignment with Burrows–Wheeler transform. Bioinformatics.

[9] Li H (2016). Minimap and miniasm: fast mapping and de novo assembly for noisy long sequences. Bioinformatics.

[10] Myers G, Brown D, Morgenstern B (2014). Efficient local alignment discovery amongst noisy long reads. Algorithms in Bioinformatics.

[11] Turakhia Y, Bejerano G, Dally WJ (2018). Darwin: A genomics co-processor provides up to 15,000x acceleration on long read assembly. SIGPLAN Not.

[12] Li H (2009). The sequence alignment/map format and samtools. Bioinformatics.

[13] Picard toolkit. Broad Institute, GitHub repository. 2019. http://broadinstitute.github.io/picard/. Accessed 11 Apr 2019.

[14] Tarasov A, Vilella AJ, Cuppen E, Nijman IJ, Prins P (2015). Sambamba: fast processing of ngs alignment formats. Bioinformatics.

[15] Faust GG, Hall IM (2014). Samblaster: fast duplicate marking and structural variant read extraction. Bioinformatics.

[16] Koboldt DC, Zhang Q, Larson DE, Shen D, McLellan MD, Lin L, Miller CA, Mardis ER, Ding L, Wilson RK (2012). VarScan 2: Somatic mutation and copy number alteration discovery in cancer by exome sequencing. Genome Res.

[17] Lai Z, Markovets A, Ahdesmaki M, Chapman B, Hofmann O, McEwen R, Johnson J, Dougherty B, Barrett JC, Dry JR (2016). VarDict: a novel and versatile variant caller for next-generation sequencing in cancer research. Nucleic Acids Res.

[18] Cibulskis K, Lawrence MS, Carter SL, Sivachenko A, Jaffe D, Sougnez C, Gabriel S, Meyerson M, Lander ES, Getz G (2013). Sensitive detection of somatic point mutations in impure and heterogeneous cancer samples. Nat Biotechnol.

[19] Garrison E, Marth G. Haplotype-based variant detection from short-read sequencing. 2012. http://arxiv.org/abs/arXiv:1207.3907. Accessed 11 Apr 2019.

[20] Wei Z, Wang W, Hu P, Lyon GJ, Hakonarson H (2011). SNVer: a statistical tool for variant calling in analysis of pooled or individual next-generation sequencing data. Nucleic Acids Res.

[21] Wilm A, Aw PPK, Bertrand D, Yeo GHT, Ong SH, Wong CH, Khor CC, Petric R, Hibberd ML, Nagarajan N (2012). LoFreq: a sequence-quality aware, ultra-sensitive variant caller for uncovering cell-population heterogeneity from high-throughput sequencing datasets. Nucleic Acids Res.

[22] Dunn T, Berry G, Emig-Agius D, Jiang Y, Lei S, Iyer A, Udar N, Chuang H-Y, Hegarty J, Dickover M, Klotzle B, Robbins J, Bibikova M, Peeters M, Strömberg M (2018). Pisces: an accurate and versatile variant caller for somatic and germline next-generation sequencing data. Bioinformatics.

[23] Kim S, Scheffler K, Halpern AL, Bekritsky MA, Noh E, Källberg M, Chen X, Kim Y, Beyter D, Krusche P, Saunders CT (2018). Strelka2: fast and accurate calling of germline and somatic variants. Nat Methods.

[24] Poplin R, Chang P-C, Alexander D, Schwartz S, Colthurst T, Ku A, Newburger D, Dijamco J, Nguyen N, Afshar PT, Gross SS, Dorfman L, McLean CY, DePristo MA (2018). A universal snp and small-indel variant caller using deep neural networks. Nat Biotechnol.

[25] Diao Y, Roy A, Bloom T. Building Highly-Optimized, Low-Latency Pipelines for Genomic Data Analysis. In: CIDR: 2015.

[26] Wong H-P, Raoux S, Kim S, Liang J, Reifenberg JP, Rajendran B, Asheghi M, Goodson KE (2010). Phase change memory. Proc IEEE.

[27] Burr G, J. Breitwisch M, Franceschini M, Garetto D, Gopalakrishnan K, Jackson B, Kurdi B, Lam C, A. Lastras L, Padilla A, Rajendran B, Raoux S, S. Shenoy R. Phase change memory technology. J Vac Sci Technol B Microelectron Nanometer Struct Process Meas Phenom Off J Am Vac Soc. 2010; 28. 10.1116/1.3301579.

[28] Condit J, Nightingale EB, Frost C, Ipek E, Lee B, Burger D, Coetzee D (2009). Better i/o through byte-addressable, persistent memory. Proceedings of the ACM SIGOPS 22Nd Symposium on Operating Systems Principles. SOSP ’09.

[29] Broad Institute. Genome Analysis Toolkit. 2010. https://software.broadinstitute.org/gatk/. Accessed 11 Apr 2019.

[30] The SAM/BAM Format Specification Working Group. Sequence Alignment/Map Format Specification. 2010. https://samtools.github.io/hts-specs/SAMv1.pdf. Accessed 11 Apr 2019.

[31] Dean J, Ghemawat S (2008). Mapreduce: Simplified data processing on large clusters. Commun ACM.

[32] McKenna A, Hanna M, Banks E, Sivachenko A, Cibulskis K, Kernytsky A, Garimella K, Altshuler D, Gabriel S, Daly M, DePristo MA. The genome analysis toolkit: a mapreduce framework for analyzing next-generation dna sequencing data. Genome Res. 2010. 10.1101/gr.107524.110. PMC2928508 20644199

[33] Broad Institute. GATK Best Practices Workflows. 2010. https://github.com/gatk-workflows. Accessed 11 Apr 2019.

[34] Institute B. GATK Variant Calling Pipelines. https://software.broadinstitute.org/gatk/best-practices/.

[35] Consortium IHGS (2004). Finishing the euchromatic sequence of the human genome. Nature.

[36] Gurdasani D, Sandhu MS, Porter T, Pollard MO, Mentzer AJ (2018). Long reads: their purpose and place. Hum Mol Genet.

[37] Apache. Apache Arrow: A Cross-language Development Platform for In-memory Data. 2019. https://arrow.apache.org/. Accessed 29 Dec 2019.

[38] Peltenburg J, van Straten J, Brobbel M, Hofstee HP, Al-Ars Z, Hochberger C, Nelson B, Koch A, Woods R, Diniz P (2019). Supporting columnar in-memory formats on fpga: The hardware design of fletcher for apache arrow. Applied Reconfigurable Computing.

[39] Apache. Plasma In-Memory Object Store. 2019. https://arrow.apache.org/blog/2017/08/08/plasma-in-memory-object-store/. Accessed 29 Dec 2019.

[40] Ahmad T, Peltenburg J, Ahmed N, Al Ars Z. Arrowsam: In-memory genomics data processing through apache arrow framework. 2019. 10.1101/741843.

[41] Herzeel C, Costanza P, Decap D, Fostier J, Verachtert W (2019). elPrep 4: A multithreaded framework for sequence analysis. PLOS ONE.

[42] Illumina. Illumina Cambridge Ltd. 2012. http://ftp.1000genomes.ebi.ac.uk/vol1/ftp/phase3/data/NA12878/sequence_read/. Accessed 24 May 2019.

[43] Apache. Apache Spark: Lightning-fast Unified Analytics Engine. 2019. https://spark.apache.org/. Accessed 2 Apr 2019.

[44] Mushtaq H, Liu F, Costa C, Liu G, Hofstee P, Al-Ars Z (2017). Sparkga: A spark framework for cost effective, fast and accurate dna analysis at scale. Proceedings of the 8th ACM International Conference on Bioinformatics, Computational Biology,and Health Informatics. ACM-BCB ’17.

[45] Massie M, Nothaft F, Hartl C, Kozanitis C, Schumacher A, Joseph AD, Patterson DA. ADAM: Genomics formats and processing patterns for cloud scale computing. Technical report, UCB/EECS-2013-207, EECS Department, University of California, Berkeley. 2013.

[46] Wang S, Yang W, Zhang X, Yu R. Performance evaluation of imp: A rapid secondary analysis pipeline for ngs data: 2018. p. 1170–6. 10.1109/BIBM.2018.8621573.

[47] Freed DN, Aldana R, Weber JA, Edwards JS. The sentieon genomics tools - a fast and accurate solution to variant calling from next-generation sequence data. 2017. 10.1101/115717.

[48] Herzeel C, Costanza P, Decap D, Fostier J, Reumers J (2015). elPrep: High-performance preparation of sequence alignment/map files for variant calling. PLOS ONE.

[49] Becker M, Chabbi M, Warnat-Herresthal S, Klee K, Schulte-Schrepping J, Biernat P, Guenther P, Bassler K, Craig R, Schultze H, Singhal S, Ulas T, Schultze JL. Memory-driven computing accelerates genomic data processing. 2019. 10.1101/519579.

[50] ApacheFoundation. Python library for Apache Arrow. 2019. https://pypi.org/project/pyarrow/. Accessed 29 Dec 2019.

[51] Shanshan R, Koen B, Zaid Al-Ars. Efficient Acceleration of the Pair-HMMs Forward Algorithm for GATK HaplotypeCaller on Graphics Processing Units. Evol Bioinforma. 2018; 14. 10.1177/1176934318760543. PMC5858735 29568218

[52] Ernst JH, Vlad-Mihai S, Koen B, Zaid Al-Ars (2018). Hardware acceleration of BWA-MEM genomic short read mapping for longer read lengths. Computa Biol Chem.

